# Using Machine Learning to Unravel the Value of Radiographic Features for the Classification of Bone Tumors

**DOI:** 10.1155/2021/8811056

**Published:** 2021-03-11

**Authors:** Derun Pan, Renyi Liu, Bowen Zheng, Jianxiang Yuan, Hui Zeng, Zilong He, Zhendong Luo, Genggeng Qin, Weiguo Chen

**Affiliations:** ^1^Department of Radiology, Nanfang Hospital, Southern Medical University, Guangzhou, Guangdong Province, China; ^2^Department of Radiology, Foshan Hospital of TCM, Foshan, Guangdong Province, China; ^3^Department of Radiology, University of Hong Kong-Shenzhen Hospital, Shenzhen, Guangdong Province, China

## Abstract

**Objectives:**

To build and validate random forest (RF) models for the classification of bone tumors based on the conventional radiographic features of the lesion and patients' clinical characteristics, and identify the most essential features for the classification of bone tumors.

**Materials and Methods:**

In this retrospective study, 796 patients (benign bone tumors: 412 cases, malignant bone tumors: 215 cases, intermediate bone tumors: 169 cases) with pathologically confirmed bone tumors from Nanfang Hospital of Southern Medical University, Foshan Hospital of TCM, and University of Hong Kong-Shenzhen Hospital were enrolled. RF models were built to classify tumors as benign, malignant, or intermediate based on conventional radiographic features and potentially relevant clinical characteristics extracted by three musculoskeletal radiologists with ten years of experience. SHapley Additive exPlanations (SHAP) was used to identify the most essential features for the classification of bone tumors. The diagnostic performance of the RF models was quantified using receiver operating characteristic (ROC) curves.

**Results:**

The features extracted by the three radiologists had a satisfactory agreement and the minimum intraclass correlation coefficient (ICC) was 0.761 (CI: 0.686-0.824, *P* < .001). The binary and tertiary models were built to classify tumors as benign, malignant, or intermediate based on the imaging and clinical features from 627 and 796 patients. The AUC of the binary (19 variables) and tertiary (22 variables) models were 0.97 and 0.94, respectively. The accuracy of binary and tertiary models were 94.71% and 82.77%, respectively. In descending order, the most important features influencing classification in the binary model were margin, cortex involvement, and the pattern of bone destruction, and the most important features in the tertiary model were margin, high-density components, and cortex involvement.

**Conclusions:**

This study developed interpretable models to classify bone tumors with great performance. These should allow radiographers to identify imaging features that are important for the classification of bone tumors in the clinical setting.

## 1. Introduction

The bone tumor is relatively rare, but the malignant bone tumor is the third leading cause of cancer-related death in individuals before 20 years old. In the United States, in 2020, an estimated 3,600 individuals (2,120 males, 1,480 females) will be diagnosed with primary malignant tumors of the bone and joints, and 1,720 individuals (1000 males, 720 females) will die from the disease [1].

The fourth edition of the World Health Organization (WHO) Classification of Tumours of Soft Tissue and Bone published in 2013 classifies bone tumors as benign, malignant, and intermediate [2]. Compared with the third edition, the most significant change is the addition of intermediate bone tumors. Intermediate bone tumors include the locally aggressive type and occasional metastatic type. Locally aggressive type often has a recurrence after resection, which is typical of osteoblastoma [2, 3]. Occasionally, metastatic type has the ability of distant metastasis, which is typically represented by giant cell tumors of bone [4]. However, the aggression and metastasis degree is lower than that of malignant bone tumors. Therefore, this classification method can better guide the formulation of clinical treatment plans. In clinical practice, bone tumor classification involves a comprehensive evaluation of a patient's demographics, medical history, and the lesion's imaging features [5]. There are significant differences in the treatment of different bone tumors; hence, the early classification of bone tumors helps guide therapy and improve patient management [6–9].

Conventional radiography is the preferred imaging modality for evaluating primary bone tumors [10]. Although the benefits of early classification of bone tumors are widely acknowledged, differentiating between bone tumor types can be difficult. Challenges include the variation in the imaging manifestation and their rarity, making it difficult for radiologists to make an accurate diagnosis [2]. Several studies have classified benign and malignant bone tumors based on patient characteristics such as age, gender, and imaging features such as tumor location, margins, periosteal reaction, and mineralization [11, 12]. Despite these efforts, no single radiographic criteria for bone tumor classification have been identified, increasing the risk for diagnostic error.

Machine learning refers to models designed to evaluate and make predictions about relationships between data [13, 14]. Classifying bone tumors using machine learning models based on predefined radiographic or clinical features may help radiologists differentiate between various bone tumors.

A random forest model is an ensemble classifier that consists of many decision trees [15]. The random forest model outputs the class voted by a majority of the individual trees or the mean individual tree prediction [16]. It generates an internal unbiased estimate of the generalization error in the forest building processes and uses a nodes' splitting process to estimate the essential variables [17]. Random forest models are highly predictive as classifiers when analyzing medical imaging data [18, 19].

We hypothesize that a random forest model with high predictive accuracy for bone tumor classification may benefit the clinical setting. This study's objectives were to (1) build and validate a random forest model to classify bone tumors based on the conventional radiographic features of the lesion and patients' clinical characteristics and (2) identify the most important conventional radiographic features for the bone tumor classification.

## 2. Materials and Method

This retrospective study was approved by the research ethics review board of Nanfang Hospital of Southern Medical University. The necessity to obtain written informed consent from included patients was waived. Data was collected by Nanfang Hospital of Southern Medical, Foshan Hospital of TCM, and University of Hong Kong-Shenzhen Hospital.

### 2.1. Study Population

The study collected 796 patients (26 ± 18 years) with pathologically confirmed bone tumors from Nanfang Hospital of Southern Medical University between 2014 and 2019, Foshan Hospital of TCM, and University of Hong Kong-Shenzhen Hospital between 2018 and 2019 as a data set. The inclusion criteria were as follows: (1) patients who underwent at least one preoperative conventional radiographic examination in one of the three academic medical centers between 2014 and 2019 and (2) patients who had a pathological diagnosis via biopsy. The exclusion criteria were as follows: (1) patients who relapse after surgery, (2) patients with poor quality preoperative conventional radiographic images, and (3) there is a foreign body in the conventional radiographic images.

For each included patient, the first preoperative conventional radiographic examination was defined as the index examination.

### 2.2. Conventional Radiography

All conventional radiographic images were collected from the picture archiving and communication system (PACS) of three hospitals. Anteroposterior and lateral views showing the bone tumor were obtained from each included patient.

### 2.3. Feature Analysis

Preoperative conventional radiographic features and potentially relevant clinical characteristics were extracted and compiled in a structured database by three musculoskeletal radiologists (with ten years of experience) without knowledge of pathological diagnoses. PACS was used to capture conventional radiographic features of each bone tumor, including location, margin, eccentric growth, expansive growth, sclerotic border, periosteal reaction, radiographic density, high-density components, the pattern of bone destruction, source, pathological fracture, and cortex involvement. The radiologists independently extracted features from the conventional radiographic images in DICOM format. Medical records were reviewed for patients' clinical characteristics, including erythrocyte sedimentation rate (ESR), age, gender, redness and hyperemia, swelling, warmth, pain, palpable mass, and dyskinesia (Table 1).

The radiologists independently scored each conventional radiographic feature, and scores were averaged across radiologists. The presence/absence of nominal features was scored on a scale from 0 to 1, where 0 indicated none of the radiologists had a positive opinion and 1 indicated all three radiologists had a positive opinion. For example, if 2 of 3 radiologists consider the margin of the bone tumor to be “sharp,” whereas the remaining 1 of 3 radiologists considered it to be “ill-defined,” the score was sharp = 0.67 (2/3) and ill-defined = 0.33 (1/3). Age and ESR were assigned numerical values.

### 2.4. Random Forest Classifier

Patients were randomly divided into a 70% training and validation data set and a 30% testing data set. A 6-fold cross-validation method was used to establish random forest models and verify the classification accuracy. The study used recursive feature elimination (RFE) to select features related to the classification during training, which enables feature interaction. RFE returns a ranking of all features by recursively training random forest models and removing the feature with the smallest ranking score. At each iteration, the feature's removal least affects the objective function. The iterations continued until the best performance of models was reached.

A binary model was built to classify tumors as benign or malignant based on the imaging and clinical data from 627 patients. The training and validation set included data from 438 patients. The test set included data from 189 patients. A tertiary model was built to classify tumors as benign, malignant, or intermediate based on the imaging and clinical data from 796 patients. The training and validation set consisted of data from 557 patients. The test set included data from 239 patients.

SHapley Additive exPlanations (SHAP) was used to describe the most important conventional radiographic features for the classification. The diagnostic performance of the random forest classifiers was evaluated in the test sets using area under curve (AUC), accuracy, sensitivity, and specificity.

### 2.5. Statistical Analysis

Statistical analysis was conducted using the SPSS version 20.0 software (SPSS, Chicago, Ill). Clinical variables were compared among patients with benign, malignant, and intermediate bone tumors using one-way analysis of variance (ANOVA). The intraclass correlation coefficient (ICC) was used to assess three radiologists' agreement who extracted radiographic features. The weights of all input variables were calculated during training and verification; the higher value of the weight indicates the greater importance. Statistical significance was set at *P* < 0.05.

## 3. Results

### 3.1. Study Population

The study enrolled 412 patients with benign bone tumors (23 ± 16 years), 215 patients with malignant bone tumors (33 ± 20 years), and 169 patients with intermediate bone tumors (24 ± 16 years). The most commonly benign, malignant, and intermediate bone tumors were osteochondroma (36.1%), osteosarcoma (45.5%), and giant cell tumor (38.5%), respectively.

For the binary classification model, the training and validation set (*n* = 438; 26 ± 18 years) consisted of 298 patients (68.0%) with a benign bone tumor and 140 (32.0%) patients with a malignant bone tumor. The test set (*n* = 189, mean age, 27 ± 18 years) consisted of 114 patients (60.3%) with a benign bone tumor and 75 (39.7%) patients with a malignant bone tumor. For the tertiary classification model, the training and validation set (*n* = 557; 26 ± 18 years) consisted of 289 (51.9%) patients with a benign bone tumor, 118 (21.2%) patients with an intermediate bone tumor, and 150 (26.9%) patients with a malignant bone tumor. The test set (*n* = 239; mean age, 26 ± 18 years) consisted of 123 (51.5%) patients with a benign bone tumor, 51 (21.3%) patients with an intermediate bone tumor, and 65 (27.2%) patients with a malignant bone tumor (Table 2). The details of the tertiary model's test set were shown in the supplement section (available here).

The clinical characteristics of the included patients stratified by bone tumor type (benign, intermediate, or malignant bone tumor) were summarized in Table 3. Patients with a malignant bone tumor were significantly older than those with a benign bone tumor (33 vs. 23 years old; *P* < 0.001). The pathological type of bone tumor was significantly associated with all clinical parameters examined except gender (*P* > 0.05).

### 3.2. Radiographic Features

The ICC agreement of three radiologists for feature extraction was high (ICC >0.75), with the lowest 0.761 for components (CI: 0.686-0.824, *P* < .001) and the highest 0.954 for location (CI: 0.936-0.967, *P* < .001), as per Table 1.

**Figure 1:**
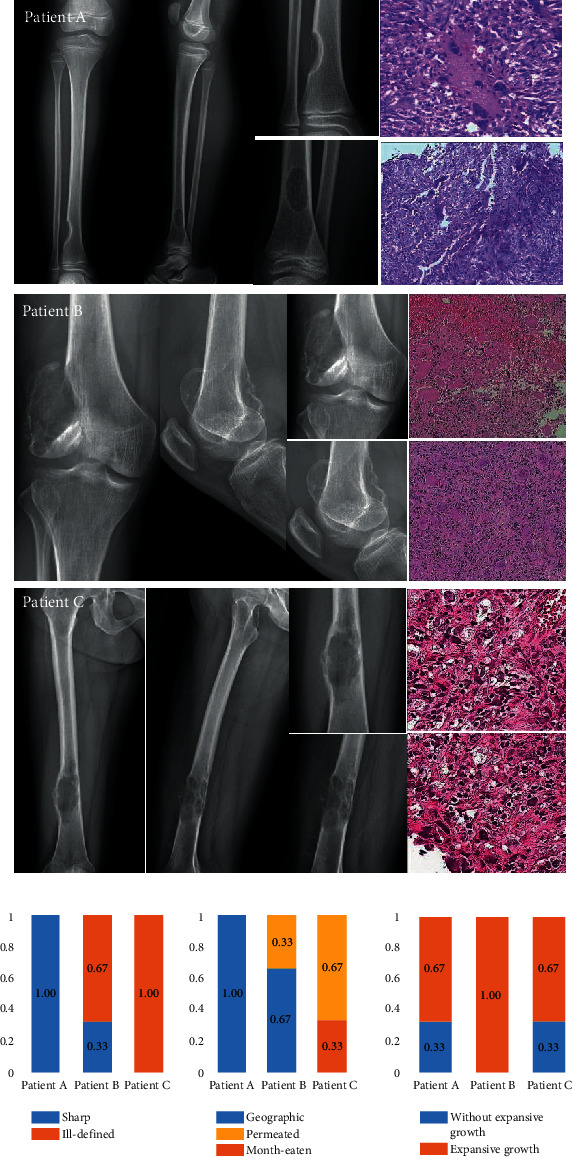
Examples of the features (upper panel) and scores depicting the presence or absence of sharp vs. ill-defined bone margins, geographic vs. moth-eaten vs. permeated pattern of bone destruction, and with vs. without expansive growth (lower panel) as seen on conventional radiographic images obtained from 3 patients. Patient A was an 8-year-old female with nonossifying fibroma. Patient B was a 34-year-old man with a giant cell tumor of bone, and Patient C was a 46-year-old woman with osteosarcoma.

Examples of the conventional radiographic features of bone tumors and their scores from 3 patients are shown in Figure 1. Patient A was an 8-year-old female with a benign bone tumor. Patient B was a 34-year-old man with an intermediate bone tumor. Patient C was a 46-year-old man with a malignant bone tumor. Images were scored for the presence or absence of sharp vs. ill-defined margins, geographic vs. moth-eaten vs. permeated pattern of bone destruction, and with vs. without expansive growth.

### 3.3. Random Forest Models

**Figure 2:**
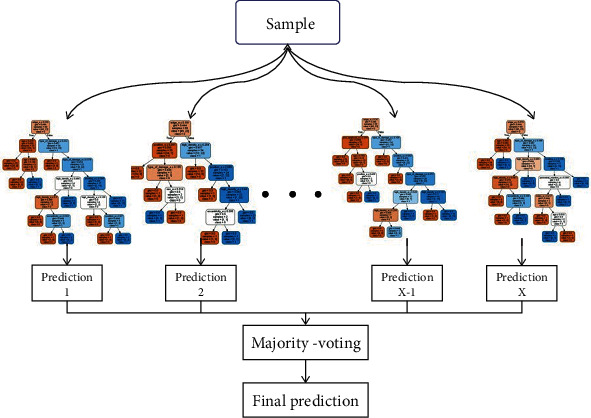
Flow chart of the random forest models. The binary classification model consisted of an ensemble of 15 random decision trees and maximum depth set to 10. The tertiary classification model consisted of an ensemble of 85 random decision trees and maximum depth set to 8. Output from all decision trees determines the final prediction.

Two random forest models were used to classify bone tumors based on imaging and clinical data (Figure 2). The binary classification model consisted of 15 random decision trees and the maximum tree depth was 10. The tertiary classification model consisted of 85 random decision trees and the maximum tree depth was 8.

The binary classification model classified bone tumors as benign or malignant. The 19 predictor variables included age, location, ESR, margin, cortex involvement, the pattern of bone destruction, high-density components, radiographic density, source, eccentric growth, gender, swelling, warmth, pain, dyskinesia, sclerotic border, location relationship with epiphysis, periosteal reaction, and pathological fracture.

The tertiary classification model classified bone tumors as benign, malignant, or intermediate. The 22 predictor variables included all the extracted conventional radiographic features and clinical characteristics.

**Figure 3:**
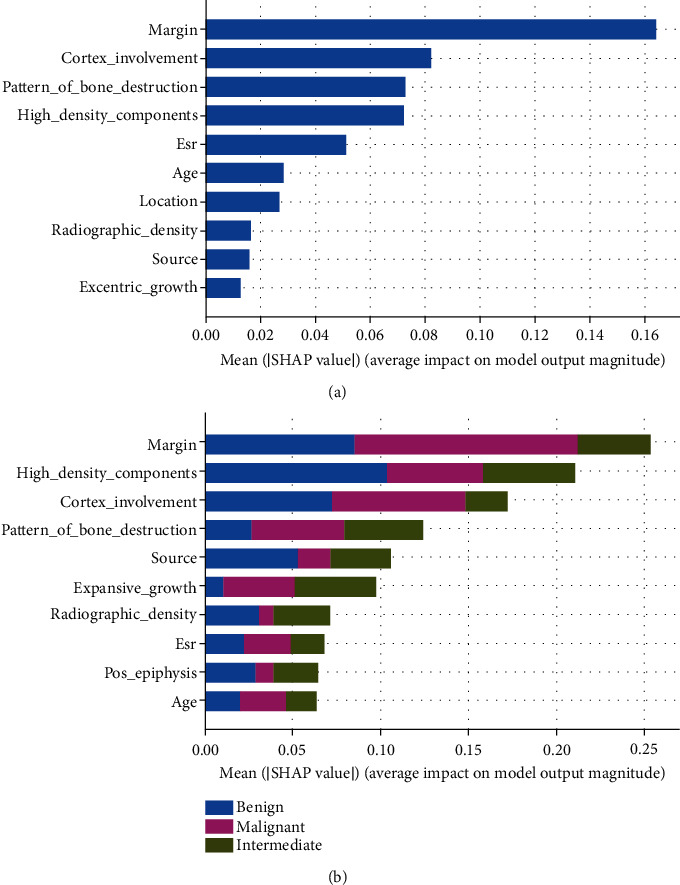
The most ten important features influencing the classification of bone tumors in the binary (a) and tertiary (b) models. The features are presented in descending order according to their absolute impact on the classification of bone tumor. The SHAP model takes into account all possible combinations of features in the presence/absence of a specific feature to evaluate its contribution to the prediction.

In descending order of importance, the binary model features were as follows: margin, cortex involvement, the pattern of bone destruction, and high-density components. The important features for the tertiary model were as follows: margin, high-density components, cortex involvement, and pattern of bone destruction (Figure 3).

### 3.4. Random Forest Model Performance

**Figure 4:**
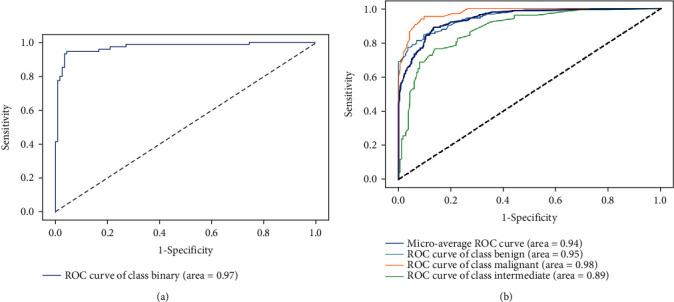
Receiver operating characteristic (ROC) curves of the binary (a) and tertiary (b) models.

The random forest models were tested for their ability to classify bone tumors as benign, malignant, or intermediate (Table 4). Overall, the binary classification model outperformed the tertiary classification model. For the binary classification model, AUC, accuracy, sensitivity, and specificity were 0.97, 94.71%, 93.33%, and 95.61%, respectively. For the tertiary classification model, AUC, accuracy, sensitivity and specificity were 0.95, 84.94%, 86.18%, and 83.62%, respectively, for predicting benign bone tumor; 0.98, 92.05%, 90.77%, and 92.53%, respectively, for predicting malignant bone tumor, and 0.89, 86.19%, 58.82%, and 93.62%, respectively, for predicting intermediate bone tumor.  Figure 4 shows the receiver operating characteristic curves for the random forest models.

## 4. Discussion

This study built, validated, and tested random forest models for the bone tumors classification based on the lesion's conventional radiographic features and patients' clinical characteristics and identified the most important conventional radiographic features for bone tumors classification. A random forest model with high performance for bone tumors classification will have utility in the clinical setting.

In this study, the most important features influencing the binary classification model were margin, cortex involvement, pattern of bone destruction, and high-density components, indicating that malignant bone tumors were more destructive and aggressive than benign bone tumors. Consistent with these results, previous reports indicate that conventional radiographic features such as lesion margins, cortical destruction, presence and type of periosteal reaction, and matrix mineralization can be applied in differentiating benign from malignant bone tumors [20, 21]. However, these studies failed to quantify which feature was more important. Regarding imaging features, the margin is considered the most critical reflection of a primary bone tumor's malignant or benign nature. Malignant tumors typically manifest as ill-defined and indistinct margins with a broad transition zone between the tumor and normal bone, while benign tumors exhibit a sclerotic rim and a narrow transition zone. In terms of high-density components, malignant bone tumors such as osteosarcoma usually include more calcified and ossified components than benign bone tumors [20]. However, some malignant tumors, including Ewing sarcoma and plasmacytoma, did not show this feature in the present study.

As for the tertiary classification model, the most important features were margin, high-density components, cortex involvement, and pattern of bone destruction. Overall, these findings support the hypothesis that an interpretable model based on conventional radiographic features and clinical characteristics can be reliably applied to classify bone tumors in clinical practice.

The binary and tertiary classification models' performances were evaluated in the test sets using AUC value, accuracy, sensitivity, and specificity. The tertiary classification model relied on more features than the binary classification model to learn and predict, while the binary model was more accurate than the tertiary model. This may be because some imaging features of intermediate bone tumors are similar to those of benign or malignant bone tumors. For example, giant cell tumor of bone appears as an eccentric lytic lesion without marginal sclerosis and may have cortical destruction on radiography [22, 23], and eosinophilic granuloma of the bone appears as a moth-eaten lytic-bone lesion without marginal sclerosis, but with a continuous periosteal reaction [24]. Retrospective analysis of misclassified cases in this study revealed that 92.3% of misclassifications involved benign vs. intermediate bone tumors or malignant vs. intermediate bone tumors.

Applying machine learning to classify bone tumors is scarce in the current study, probably because of bone tumor's rareness, variable location, and appearance, making data collection a challenge. Benndorf et al. built a pretest probabilistic (naive Bayes) classifier for primary malignant bone tumors based on the patient's age, sex, and tumor localization. Results from ten-fold cross-validation showed that the pretest probability of primary malignant bone tumor was correctly raised in 79.8% of cases [25]. Do et al. used a naive Bayes machine that processed 18 demographic and radiographic features to evaluate primary and differential accuracy for the diagnosis of bone tumors. Primary accuracy was 62% and differential accuracy was 80% for the top 10 most common diagnoses [26]. In the present study, the binary and tertiary classification models' accuracy was 94.71% and 82.77%, indicating that these random forests outperformed previously reported models with superior accuracy. Unlike the previous study, this study evaluated model performance using AUC, which is more suitable for medical bias data.

The random forest model with reliable classification performance may assist radiologists in bone tumor diagnosis. Misdiagnosis and inappropriate treatment can also be reduced to a certain extent. It can improve the cure rate and prognosis of patients with bone tumors to a great extent eventually.

To the author's knowledge, the present study is the first to identify the most important conventional radiographic features for the bone tumor classification [27–29]. Thirteen conventional radiographic features were used to distinguish among benign, malignant, and intermediate bone tumors. Data from three medical centers were used to train, validate, and test the models, implying that the models are widely applicable across various clinical settings. This contrasts with other approaches based on image analysis, such as radiomics, which can be limited by different healthcare institutions' scanner parameters and image processing software [30, 31].

There are several limitations to this study. First, the classification models were based on conventional radiographic features without considering other imaging modalities such as computed tomography (CT) and magnetic resonance imaging (MRI). Thus, some imaging features that are important for the classification may have been missed. However, conventional radiography is the preferred imaging modality for evaluating primary bone tumors. Therefore, models based on conventional radiographic features provide suitable and convenient solutions to guide clinical decision-making in bone tumor classification. Second, some patients' clinical characteristics were incomplete, and several specific biochemical markers of bone tumors, such as alkaline phosphatase, were not collected.

In conclusion, our study developed binary and tertiary models trained on a data set of linked conventional radiographic features and clinical characteristics to classify bone tumors, which obtained outstanding performance. Unlike previous studies, the SHapley Additive exPlanations was used to help radiologists, and other physicians recognize imaging features that are important for bone tumor classification. This approach may allow doctors to understand models easily so that they can integrate it into clinical practice to make precise diagnoses. In the future, the models may be enhanced by integrating CT and MRI features, potentially improving bone tumor classification and patient outcomes.

## Figures and Tables

**Table 1:** Preoperative radiographic features and clinical characteristics with potential clinical importance for diagnosis.

Features	Feature class	Permissible value	ICC
Location^∗^	Categorical	Upper tibia (a)/inferior femur (b)/upper humerus (c)/middle humerus (d)	0.954
Location	Categorical	Epiphysis (a)/metaphysis (b)/diaphysis (c)/not applicable (d)	0.854
Eccentric growth	Binary	Without (0)/with (1)	0.921
Expansive growth	Binary	Without (0)/with (1)	0.888
Margin	Binary	Sharp(0)/ill-defined (1)	0.832
Sclerotic border	Binary	Without (0)/with (1)	0.796
Periosteal reaction	Categorical	Without (a)/continuous (b)/interrupted (c)	0.899
Radiographic density	Categorical	Mixed (a)/low (b)/high (c)	0.863
High-density components	Categorical	Without (a)/calcification or ossification (b)/tumor bone (c)/unrecognizable (d)	0.761
Pattern of bone destruction	Categorical	Geographic (a)/moth-eaten (b)/permeated (c)/not applicable (d)	0.812
Source	Binary	Medullary (0)/cortical (1)	0.909
Pathological fracture	Binary	Without (0)/with (1)	0.888
Cortex involvement	Categorical	Complete cortex (a)/cortical expansion and thinning (b)/interrupted cortex (c)	0.870
Clinical data			
ESR	Numerical		—
Age	Numerical		—
Gender	Binary	Male (0)/female (1)	—
Redness and hyperemia	Binary	Without (0)/with (1)	—
Swelling	Binary	Without (0)/with (1)	—
Warmth	Binary	Without (0)/with (1)	—
Pain	Binary	Without (0)/with (1)	—
Palpable mass	Binary	Without (0)/with (1)	—
Dyskinesia	Binary	Without (0)/with (1)	—

Note: ^∗^The location details are shown in supplement section.

**Table 2:** Patients characteristics: training, validation and test sets.

Characteristics	Binary model		Tertiary model	
	Training and validation set	Test set	Training and validation set	Test set
No. of patients	438	189	557	239
Age (y)^∗^	26 ± 18	27 ± 18	26 ± 18	26 ± 18
ESR^∗^	19.01 ± 22.20	20.47 ± 24.09	19.95 ± 23.16	20.97 ± 24.70
Pathological results				
Biopsy benign for bone tumor	298 (68.0)	114 (60.3)	289 (51.9)	123 (51.5)
Biopsy malignant for bone tumor	140 (32.0)	75 (39.7)	150 (26.9)	65 (27.2)
Biopsy intermediate for bone tumor	0	0	118 (21.2)	51 (21.3)

Note: unless otherwise indicated, data are numbers (%) of patient. ^∗^Data are means ± standard deviation.

**Table 3:** Clinical characteristics of the included patients stratified by benign, intermediate, or malignant bone tumor.

Clinical characteristics	All (*N* = 796)	Benign (*N* = 412)	Malignant (*N* = 215)	Intermediate (*N* = 169)	*P* value
Age	26 ± 18	23 ± 16	33 ± 20	24 ± 16	<0.001^∗^
ESR	20.26 ± 23.63	12.25 ± 14.96	33.25 ± 28.24	23.25 ± 26.38	<0.001^∗^
Male	496 (62.3)	253 (61.4)	135 (62.8)	108 (63.9)	0.841
Female	300 (37.7)	159 (38.6)	80 (37.2)	61 (36.1)	
Redness and hyperemia	20 (2.5)	5 (1.2)	13 (6.0)	2 (1.2)	0.018^∗^
Without redness and hyperemia	776 (97.5)	407 (98.8)	202 (94.0)	167 (98.8)	
Swelling	211 (26.5)	79 (19.2)	85 (39.5)	47 (27.8)	<0.001^∗^
Without swelling	585 (73.5)	333 (80.8)	130 (60.5)	122 (72.2)	
Warmth	92 (11.6)	12 (2.9)	59 (27.4)	21 (12.4)	<0.001^∗^
Without warmth	704 (88.4)	400 (97.1)	156 (72.6)	148 (87.6)	
Pain	472 (59.3)	173 (42.0)	174 (80.9)	125 (74.0)	<0.001^∗^
Without pain	324 (40.7)	239 (58.0)	41 (19.1)	44 (26.0)	
Palpable mass	275 (34.5)	169 (41.0)	73 (34.0)	33 (19.5)	<0.001^∗^
Without palpable mass	521 (65.5)	243 (59.0)	142 (66.0)	136 (80.5)	
Dyskinesia	171 (21.5)	53 (12.9)	69 (32.1)	49 (29.0)	<0.001^∗^
Without dyskinesia	625 (78.5)	359 (87.1)	146 (67.9)	120 (71.0)	

^∗^ *P* < 0.05.

**Table 4:** The performance of random forest models.

	AUC	Sensitivity	Specificity	Accuracy	Overall accuracy	Micro AUC
Binary model	0.97	93.33%	95.61%	94.71%		
Tertiary model						
Benign	0.95	86.18%	83.62%	84.94%	82.77%	0.94
Malignant	0.98	90.77%	92.53%	92.05%		
Intermediate	0.89	58.82%	93.62%	86.19%		

Note: AUC: area under the receiver operating characteristic curve. All the results were obtained in the test set of two models.

## Data Availability

In order to protect the privacy of patients, the access to data is restricted.

## Supplementary Materials

Supplementary Materials Supplement Section: the locations represented by each letter. The type and number of tumors in the tertiary model's test set.
