# Supplementary material for: Using Machine Learning to Unravel the Value of Radiographic Features for the Classification of Bone Tumors
Source: Biomed Res Int. 2021 Mar 11;2021:8811056. doi: 10.1155/2021/8811056 (PMC7984886; doi:10.1155/2021/8811056)
Supplement: Supplementary Materials — Supplement Section: the locations represented by each letter. The type and number of tumors in the tertiary model's test set. [file 8811056.f1.pdf]

**Supplement Section:**

**The locations represented by each letter**

| Value | Location | Value | Location |
|---|---|---|---|
| a | Upper tibia | l | Middle ulna |
| b | Inferior femur | m | Distal radius |
| c | Upper humerus | n | Lumbar vertebrae |
| d | Middle humerus | o | Distal ulna |
| e | Sacrum | p | Hand |
| f | Distal humerus | q | Proximal ulna |
| g | Foot | r | Middle tibia |
| h | Pelvis | s | Scapula |
| i | Middle femur | t | Proximal fibula |
| j | Distal tibia | u | clavicle |
| k | Proximal femur | v | proximal radius |

**The type and number of bone tumors in the tertiary model's test set**

| Benign bone tumor (123) | Intermediate bone tumor (51) | Malignant bone tumor (65) |
|---|---|---|
| Non-ossifying fibroma (11) | Aneurysmal bone cyst (13) | Osteosarcoma (34) |
| Osteochondroma (49) | Giant cell tumor of bone (25) | Spindle cell malignancies (2) |
| Bone cysts (10) | Osteoblastoma (4) | Ewing's sarcoma (3) |
| Osteofibrous dyspisia (17) | Eosinophilic granuloma of bone (9) | Malignant giant cell tumor of bone (1) |
| Fibroxanthoma of bone (1) | | Chordoma (3) |
| osteoidosteoma (14) | | Plasmacytoma (2) |
| Benign fibrous histiocytoma (3) | | Lymphoma of bone (1) |
| Endogenous chondroma (15) | | Chondrosarcoma (5) |
| Neurilemmoma of bone (1) | | Bone metastases (14) |
| Hemangioma (2) | | |
